# Metabolic phenotype of breast-fed infants, and infants fed standard formula or bovine MFGM supplemented formula: a randomized controlled trial

**DOI:** 10.1038/s41598-018-36292-5

**Published:** 2019-01-23

**Authors:** Xuan He, Mariana Parenti, Tove Grip, Magnus Domellöf, Bo Lönnerdal, Olle Hernell, Niklas Timby, Carolyn M. Slupsky

**Affiliations:** 1 Department of Nutrition, University of California Davis, One Shields Ave, Davis, CA 95616 USA; 2 Department of Food Science and Technology, University of California Davis, One Shields Ave, Davis, CA 95616 USA; 3 Department of Clinical Sciences, Pediatrics, SE 901 85 Umeå University, Umeå, Sweden

**Keywords:** Metabolomics, Paediatric research

## Abstract

Formula-fed (FF) infants exhibit a different metabolic profile than breast-fed (BF) infants. Two potential mechanisms are the higher protein level in formula compared with breast milk and the removal of the milk fat and associated milk fat globule membranes (MFGM) during production of infant formula. To determine whether MFGM may impact metabolism, formula-fed infants were randomly assigned to receive either an MFGM isolate-supplemented experimental formula (EF) or a standard formula (SF) from 2 until 6 months and compared with a BF reference group. Infants consuming EF had higher levels of fatty acid oxidation products compared to infants consuming SF. Although the protein level in the study formula was approximately 12 g/L (lower than most commercial formulas), a metabolic difference between FF and BF remained such that FF infants had higher levels of amino acid catabolism by-products and a low efficiency of amino acid clearance (preference for protein metabolism). BF infants had higher levels of fatty acid oxidation products (preference for fat metabolism). These unique, energy substrate-driven metabolic outcomes did not persist after diet was shifted to weaning foods and appeared to be disrupted by complementary feeding. Our results suggest that MFGM may have a role in directing infant metabolism.

## Introduction

Breast milk is considered to be the best source of cellular fuel and essential nutrients for the rapidly growing brain of the infant. Indeed, the infant brain requires approximately 74% of the total dietary energy intake, whereas in adults, only 20–23% of dietary energy is used to nourish the brain^1^. Human infants have higher brain:body mass ratios and have more body fat than offspring of many other mammalian species. Indeed, a normally developing infant’s body fat continuously increases from 15–16% of their body weight at birth to 25%-26% of their body weight at 12–18 months of age^2^. During this time, the metabolic phenotype of the infant is geared toward compensating the high energy demands of the developing brain^3^.

Human brain development accelerates in the first few months after birth, when a temporal neurodevelopmental window paralleled by major shifts in metabolism is observed. Several studies support the benefits of breastfeeding on improving cognitive developmental outcomes^4^ and preventing overweight and obesity^5^. While human milk is the ideal source of nutrition for infants, the decision to breast-fed and its duration are highly personal and influenced by historical, socioeconomic, cultural and individual-level factors^6^. A metabolic phenotype characterized by high levels of circulating amino acids, insulin and urea has consistently been observed in formula-fed (FF) infants^7–11^. We hypothesize this is an outcome of “too much protein in infant formula and insufficient use of fat”. However, whether this is a temporal reflection of differences in nutrient intake during this specific time frame of feeding, or whether this may have profound long-lasting effects remains to be elucidated.

Reducing the protein content of formula has been shown to decrease later risk of childhood overweight and obesity at school age^12,13^. Efforts have therefore been made to successively lower the protein content of infant formula while maintaining adequate growth and plasma amino acids at levels similar to those of breast-fed infants. Milk fat globule membranes (MFGM), an important component of milk, have historically been discarded with the milk fat during production of infant formula. The MFGM contains numerous complex lipids such as phospholipids, sphingolipids and gangliosides, sialic acid and cholesterol, as well as bioactive glycoproteins such as mucin (MUC1), lactadherin, butyrophilin and xanthine oxidase^14^. Therefore, supplementing infant formula with a bovine MFGM isolate may provide an efficacious and sustainable approach to address the current nutritional and developmental gaps observed between breast-fed and formula-fed infants.

Previously, we conducted a double-blind, randomized controlled trial where 240 infants were enrolled at less than 2 months of age. All infants were, based on parental reporting, exclusively breast-fed (BF) or formula-fed (FF) prior to enrollment. The FF infants were subsequently assigned to consume either an MFGM-supplemented, low-energy, low-protein experimental formula (EF) or a standard formula (SF) from 2 until 6 months of age (baseline demographics and clinical characteristics for each group are available elsewhere^15^). To access the impact of this dietary intervention on metabolism, serum samples were collected at 2 (baseline), 4, 6 and 12 (post-intervention) months of age.

In this cohort, we reported that during the intervention period, FF infants displayed higher growth velocity compared to the BF infants^15^. This elevated growth in the FF infants was coupled with higher plasma insulin and blood urea nitrogen levels. In contrast, BF infants had higher levels of triglycerides and cholesterol in serum, as well as higher LDL:HDL ratios and leptin:fat mass ratios. Furthermore, infants who consumed EF showed a different circulating lipidomics profile^16^, as well as higher serum cholesterol and triglyceride levels compared to the SF-fed infants that approached what was observed in the BF infants^17^ (Primary and secondary outcome measures from this cohort were previously published, and are summarized in Table 1).

**Table 1:** Differences between breast-fed (BF), SF (Standard Formula) and EF (Experimental Formula) fed infants previously reported in this cohort.

		Baseline	Intervention period		Post-intervention	Ref
		2 mon of age	4 mon of age	6 mon of age	12 mon of age	
Growth parameters and clinical assessment	Weight-for-age, length-for-age, head circumference -for-age, BMI-for-age (z-score)	NS	NS	NS	NS	^15^
	Growth velocity (weight and length)	(EF + SF) > BF				^15^
	% body fat	NS	NS			^15^
	Blood pressure (systolic and diastolic)	NS	NS	NS	NS	^17^
Blood biochemical assessment	Plasma Insulin	(EF + SF) > BF	(EF + SF) > BF	(EF + SF) > BF		^15^
	Blood urea nitrogen	(EF + SF) > BF	(EF + SF) > BF	(EF + SF) > BF		^15^
	Total serum cholesterol	BF > SF, BF > EF	BF > EF > SF	BF > SF, EF > SF	NS	^17^
	Serum LDL: HDL	BF > SF, BF > EF	BF > SF, BF > EF	BF > SF, BF > EF	NS	^17^
	Serum triglyceride	NS	BF > EF > SF	NS	NS	^17^
	Serum Leptin		NS			^17^
	Serum High-molecular-weight adiponectin		NS			^17^
	Leptin: fat mass		BF > SF, BF > EF			^17^
	Serum homocysteine	BF > EF, BF > SF	BF > EF	BF > EF, SF > EF	NS	^17^
Inflammatory markers and immune development	Serum C-reactive protein			BF > EF, SF > EF	NS	^17^
	Serum IgG against pneumococci (serotypes 1, 5 and 14)			SF > EF		^67^
	Fecal calprotectin	BF > EF, BF > SF	BF > EF, BF > SF	NS		^17^
Bayley Scale of Infant and Toddler Development	Cognitive score				EF > SF	^15^
	Motor score (fine motor)				BF > SF, BF > EF	
	Verbal score (receptive)				BF > SF, BF > EF	

Footnote: NS: not significant.

Here, we further explored three aims within this cohort: (1) whether a comprehensive evaluation of the serum metabolome will reflect the effect of early diet at 12 months of age; (2) whether a metabolic signature is present that indicates improved cognitive development as previously reported in this cohort; and (3) whether consumption of a formula supplemented with a bovine MFGM isolate (EF) improves the overall metabolic outcome when compared to infants fed SF.

## Results

To expand our understanding of dietary-driven metabolic changes in early development, both plasma and serum samples from this randomized controlled trial were subjected to a comprehensive metabolomics analysis. Metabolites from the serum samples at 2, 4, 6 and 12 months of age were quantified using a targeted NMR metabolomics approach (“the longitudinal subset”) to evaluate metabolic changes by different feeding regiments over time. Plasma samples at 6 months of age were subjected to untargeted LC-MS and GC-MS analysis (“the cross-sectional subset”) with the aim of exploring the impact of early feeding on lysophosphatidylcholines (LysoPCs), acylcarnitines and free fatty acids in serum, and comparing soluble metabolites with the NMR results. Metabolites that are identified by NMR, LC-MS and GC-MS are summarized in SI Table 1.

### Diet-induced serum metabolic profile over time (“the longitudinal subset”)

**Figure 1:**
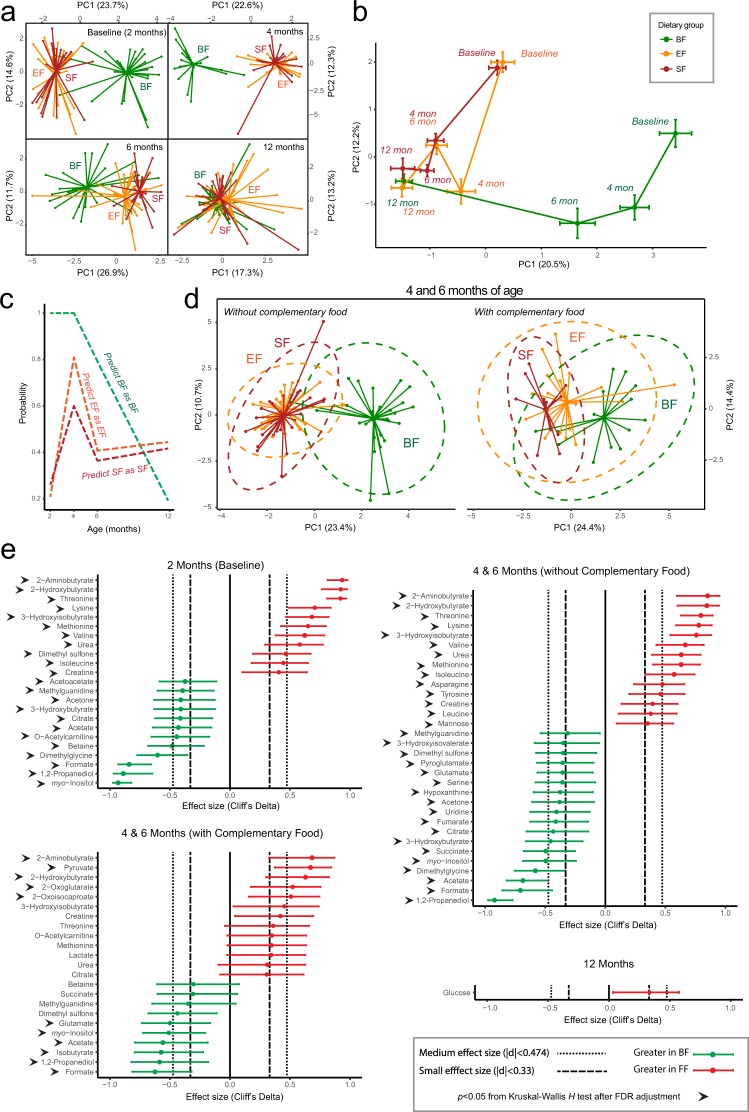
Targeted serum metabolome analysis by NMR reveals differences between breast-fed (BF, green) and formula-fed (FF) (Experimental Formula, EF, orange; Standard Formula, SF, red) infants at 2, 4 and 6 months of age. (**a**) Principal component analysis (PCA) of generalized log transformed serum metabolite concentration data. The number of samples from each group is summarized in SI Tables 2 and 3. (**b**) Shifting of centroids over time from clusters in A). The centroids and the associated error bars are calculated using the average and standard deviation of PC1 and PC2 within each cluster. (**c**) Prediction outcome from random forest analysis. (**d**) PCA of infants from 4 and 6 months of age who had no/low or relatively high consumption of complementary food. The ellipses were constructed based on multivariate normal distribution at 95% confidence level. (**e**) NMR quantified serum metabolites significantly different between BF (green) and FF (red) infants. The 95% confidence interval was estimated using a normal distribution.

Starting from 2 months of age, a difference in the serum metabolic profile between BF and FF infants was observed, which began to overlap by 6 months and eventually fully overlap when infants were 12 months of age (Fig. 1a,b). A random forest trained prediction model further confirmed this (Fig. 1c). The probability of predicting BF as BF was highest at 2 and 4 months and decreased over time as complementary foods were introduced. By 12 months, the overall metabolic profiles of BF and FF were mostly indistinguishable. To further test whether this metabolic shift was driven by the introduction of complementary food, samples collected at 4 and 6 months were divided into two subgroups depending on complementary food intake: those with (>60 kcal), or those without (<60 kcal) of daily energy from complementary food. Infants consuming little or no complementary food revealed a more profound separation upon comparison of BF and FF (Fig. 1d). Introduction of complementary food minimized this difference.

The BF infant serum metabolome was characterized by metabolites indicative of elevated ketogenesis, with increased 3-hydroxybutyrate, acetone, as well as the fat oxidation by-products acetate and formate. In contrast, the serum metabolome of FF infants was characterized by protein catabolism, showing elevated metabolites within the protein degradation pathway including the nitrogen waste product urea, essential amino acids (isoleucine, leucine, valine, tyrosine, asparagine, methionine, threonine), and amino acid catabolism by-products (2-hydroxybutyrate and 3-hydroxyisobutyrate) (Fig. 1e).

**Figure 2:**
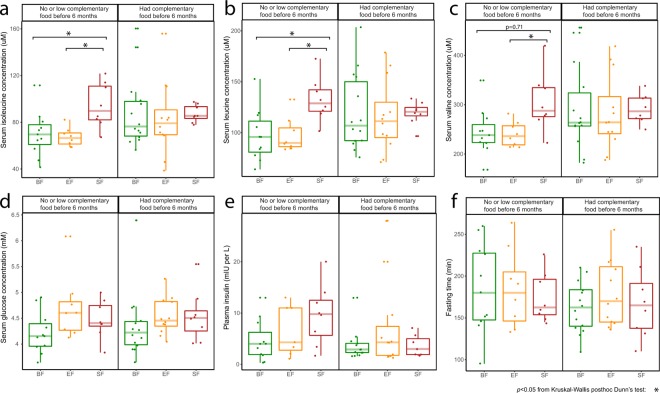
A post-intervention effect at 12 months of age was observed between the breast-fed (BF, green) and standard formula-fed (SF, red), and between the SF and experimental formula-fed (EF, orange) in NMR measurement of serum (**a**) isoleucine, (**b**) leucine, (**c**) valine, only when no or low complementary food was introduced before 6 months of age. (**d**) Serum glucose and (**e**) plasma insulin measurements (adapted from previous published work^15^) were not significantly different. (**f**) No significant difference in fasting time was observed. Significance was evaluated using Kruskal-Wallis posthoc Dunn’s test at p < 0.05.

As increasing amounts of complementary food were introduced, many of the metabolites that separated BF and FF based upon the preference of fat or protein utilization differed less or became non-significant. However, serum metabolites such as 2-aminobutyrate, 2-hydroxybutyrate, *myo*-inositol and 1,2-propanediol remained as differentiating markers separating the BF and FF groups regardless of complementary food consumption. By 12 months, the majority of serum metabolites were similar between BF and FF, except glucose, which was slightly elevated in the FF group (medium effect size, not significant by Kruskal-Wallis after FDR adjustment, Fig. 1e, Fig. 2d).

A few serum metabolites were different between the SF and EF groups. The majority of these metabolites had a medium effect size but were not statistically significant by Kruskal-Wallis (after FDR correction). A higher level of dimethyl sulfone was observed in the SF group compared to the EF group, but only when no (or little) complementary food was introduced at 4 and 6 months. Metabolites that were high in BF compared with FF, including *myo*-inositol, -acetylcarnitine (C2), acetone, 3-hydroxybutyrate, isobutyrate, also exhibited an increased trend in the EF group compared with the SF group (SI Fig. 1).

To evaluate whether complementary food introduction prior to 6 months changed the post-intervention effect at 12 months of age, a subset of data based on dietary record from 12 months of age was further examined. An elevated level of circulating branched chain amino acids (BCAA) was observed between BF and infants fed SF and between the SF and EF groups only when comparing infants who had <60 kcal of dietary energy from complementary food before 6 months of age (Fig. 2a–c). Serum glucose and plasma insulin were not different (Fig. 2d,e), and there was no difference in fasting time (Fig. 2f, SI Table 4, Kruskal-Wallis post-hoc Dunn’s test).

### Diet-induced plasma metabolic profile at 6 months of age (“the cross-sectional subset”)

**Figure 3:**
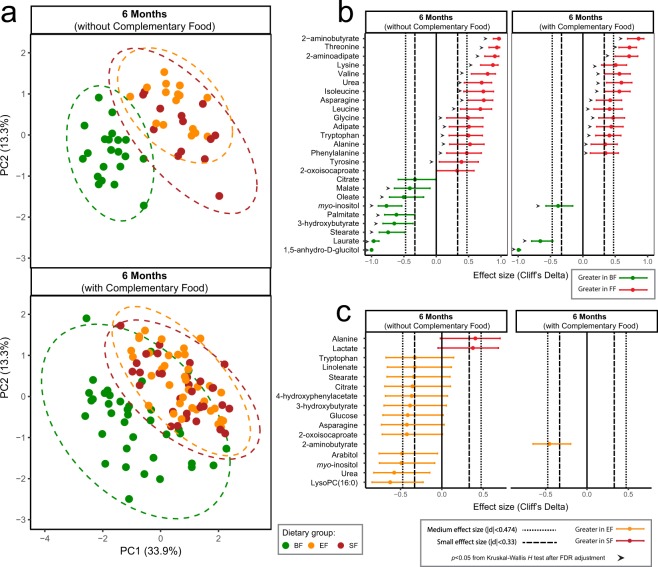
Untargeted plasma metabolome by GC-MS reveals a difference between breast-fed (BF, green) and formula-fed (Experimental Formula, EF, orange; Standard Formula, SF, red) infants at 6 months of age. (**a**) Principal component analysis (PCA) of generalized log transformed intensity data. The corresponding plasma metabolites significantly different between (**b**) BF and formula-fed, and between (**c**) SF and EF infants at 6 months of age. The 95% confidence interval was estimated using the normal distribution. The number of samples from each group is summarized in SI Table 3.

To validate the result from “the longitudinal subset” and obtain more insights into the mechanism underlying the metabolic differences between BF and FF infants with respect to changes in circulating fatty acids, acylcarnitines and LysoPCs, plasma samples obtained from infants at 6 months of age were subjected to GC-MS and LC-MS untargeted analysis. In agreement with the NMR metabolomics data, the untargeted GC-MS analysis of the plasma metabolome revealed a profound difference between BF and FF (Fig. 3a) with elevated levels of circulating *myo*-inositol and 3-hydroxybutyrate in BF, and elevated levels of circulating amino acids (threonine, lysine, BCAAs), nitrogen waste products (urea) and amino acid degradation intermediate products (2-aminobutyrate and 2-aminoadipate) in FF infants. Introduction of complementary food reduced the effect size of these differentiating metabolites detected using GC-MS between BF and FF infants (Fig. 3b), in agreement with the metabolomics findings from NMR. GC-MS data further confirmed the higher level of circulating *myo*-inositol and 3-hydroxybutyrate in the EF group as compared to the SF group, although not statistically significant by Kruskal-Wallis test after FDR adjustment (Fig. 3c).

**Figure 4:**
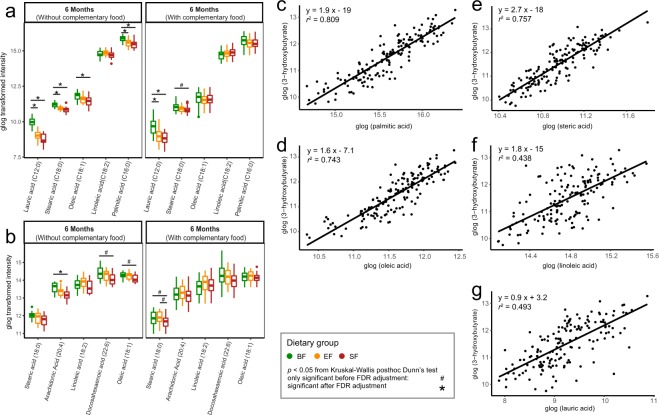
Plasma free fatty acids are different between the breast-fed (BF, green) and formula-fed (Experimental formula, EF, orange; Standard Formula, SF, red) infants. The significant differences in plasma free fatty acids were measured using (**a**) GC-MS and (**b**) LC-MS metabolomics approach and evaluated using Kruskal-Wallis posthoc Dunn’s test. Correlation between the plasma levels of 3-hydroxybutyrate and the free fatty acids, (**c**) palmitic acid, (**d**) oleic acid, (**e**) steric acid, (**f**) linoleic acid, and (**g**) lauric acid, at 6 months of age. Data for correlation analysis was quantified using GC-MS. The association coefficient was evaluated using the square of the Pearson’s correlation coefficient (*r*^2^) and visualized via linear regression.

In contrast to human milk, infant formula contains lipids that are derived from a mixture of vegetable oils, and therefore is distinctly different with regard to fatty acid composition, triglyceride structure, concentration of cholesterol, as well as fat globule size and architecture^18–20^. Higher levels of circulating triglycerides were found in BF compared to the FF infants in this cohort^17^. Through evaluation of circulating free fatty acids using GC-MS and LC-MS, several free fatty acids including lauric acid (C12:0), palmitic acid (C16:0), oleic acid (C18:1), and arachidonic acid (C20:4) were higher in BF compared with FF infants (Figs 3b and 4a,b). Among these metabolites, palmitic acid (C16:0), oleic acid (C18:1) and steric acid (C18:0) showed strong positive relationships with circulating 3-hydroxybutyrate (*r*^2^ > 0.74), whereas linoleic acid (C18:2) and lauric acid (C12:0) showed a weaker association (*r*^2^ = 0.44 and *r*^2^ = 0.50, respectively, Fig. 4c–g). The positive relationship between circulating fatty acids and 3-hydroxybutyrate provides evidence of ketone body synthesis in the liver as a consequence of fatty acid oxidation.

**Figure 5:**
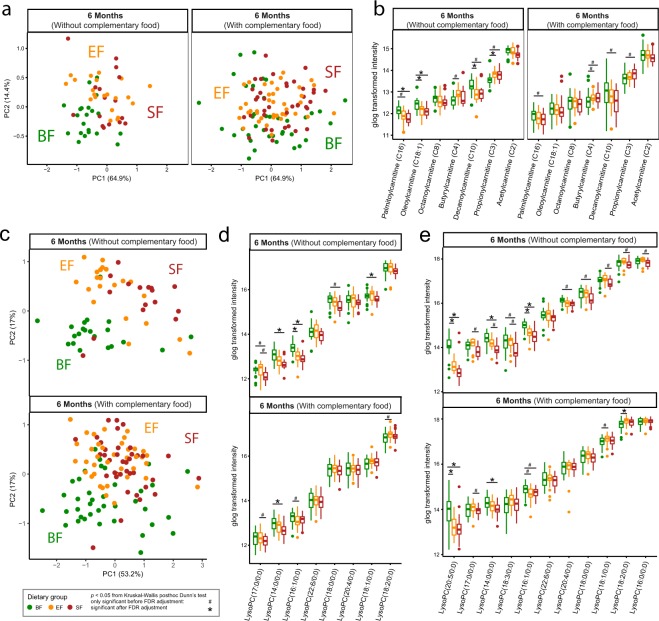
Plasma acylcarnitines and lysophophatidylcholine (LysoPC) species are different between breast-fed (BF, green) and formula-fed (Experimental formula, EF, orange; Standard Formula, SF, red) infants. (**a**) Principal component analysis (PCA) of generalized log transformed plasma acylcarnitines measured in the positive-ion mode of untargeted LC-MS. (**b**) Significantly different plasma acylcarnitine species were evaluated using Kruskal-Wallis with Dunn’s posthoc comparison. (**c**) PCA of generalized log transformed plasma LysoPC species measured in both the positive- and negative- ion mode of untargeted LC-MS. The significantly different plasma LysoPC species in (**d**) the positive- ion mode and **(e)** the negative- ion mode were evaluated using Kruskal-Wallis posthoc Dunn’s test.

Mitochondrial fatty acid beta-oxidation requires conjugation of long-chain fatty acids to a carnitine molecule (also known as acylcarnitines) to be transported across the inner mitochondrial membrane. In BF infants, a few medium- and long-chain acylcarnitine species including decanoyl-L-carnitine (C10), palmitoyl-L-carnitine (C16), and oleoyl carnitine (C18:1) were significantly higher in comparison to the FF group. The FF group showed higher levels of the short chain acylcarnitine, propionyl-carnitine (C3). This difference between acylcarnitine species became less profound when a significant amount of complementary food was consumed at and before 6 months of age (Fig. 5a,b).

Through plasma LC-MS analysis, a discrepancy between BF and FF was observed in LysoPC profile (Fig. 5c). BF infants showed higher circulating lysoPC (16:1/0:0), and lysoPC (14:0/0:0), as well as a trend toward higher levels of LysoPC (18:3/0:0) compared to FF infants. EF infants showed a trend toward higher LysoPC (14:0/0:0), (16:0/0:0), (17:0/0:0), (18:1/0:0), (18:2/0:0), and (18:3/0:0) in the plasma compared with SF infants (Fig. 5d,e). The tendency toward increased circulating LysoPC (16:0/0:0) in the EF group was also observed using GC-MS (medium effect size, not significant by Kruskal-Wallis test, after FDR (Fig. 3c)). These higher levels of circulating LysoPCs in EF were coupled with higher levels of serum choline, as well as its downstream products betaine and dimethylglycine compared with the SF infants (SI Fig. 1).

## Discussion

### Preference for fat-based energy metabolism in breast-fed infants

We previously reported higher fasting levels of circulating triglycerides in BF compared with FF infants^17^ (Table 1). Since the mobilization of body fat is regulated by insulin, lower plasma insulin levels previously observed in human (Table 1, and others^21^) and rhesus^22^ BF infants suggest an increased rate of lipolysis in adipose tissue as well as fatty acid oxidation in muscle and liver in BF infants compared with FF infants. Circulating non-esterified fatty acids (NEFA) reflect adipose output and storage, being higher after fasting, and decreasing in the post-prandial state when NEFA release from adipose tissue is suppressed. Circulating lipids are more rapidly cleared in infants as their post-meal triglyceride levels return to baseline within 180 min in an infant, compared to 8 h in adults^23^. We previously showed that at 120 min post-meal, plasma NEFA levels of infants were similar to pre-prandial (~3 h of fasting) levels^9^. Therefore, the profile of circulating fatty acids and triglycerides reported here (at approximately 165 min post-meal, SI Table 4) reflects fasting conditions. In this study, we observed higher circulating fatty acids (C12:0, C16:0, C18:0, C18:1, and C20:4, from Figs 3b and 4a,b) and medium- and long-chain acylcarnitine species (C10, C16, C18:1) (Fig. 5b) in BF infants. Kirchberg *et al*. also observed higher long-chain acylcarnitine (C14, C16 and C18) to free carnitine ratios in the plasma of BF infants, with lower levels found in infants consuming a higher fat, lower protein formula, and the lowest levels in infants consuming a lower fat, higher protein formula^10^. Interestingly, higher circulating triglycerides were also reported in exclusively BF compared with mixed-fed infants^24^.

The higher serum levels of long- and medium- chain fatty acids, along with increased levels of acylcarnitine species (derived from skeletal muscles and other tissues such as liver and cardiac muscles), are suggestive of fatty acid oxidation flux. The levels of acetate and formate, as products of fatty acid oxidation^25^, were higher in the serum of BF infants before 6 months of age regardless of introduction of complementary food (Fig. 1e). This observation is consistent with our previous metabolomics work on breast-fed infants in both the semi-fasting and postprandial states^9^. With respect to circulating acetate, the activity of the milk oligosaccharide fermenting gut microbiota, especially the *Bifidobacterium* species which produce acetate^26,27^, must also be considered. However, further studies are needed to address any possible relationship between serum acetate and gut microbial activity.

Higher circulating levels of ketone bodies were also observed in BF infants compared with FF infants in the present study when no or a limited amount complementary food was consumed (Figs 1e, 3b), in line with previous observations in other human infant studies^21,28^ as well as in rhesus macaque infants^22^. We believe that this represents a unique case of ketone production when glucose is sufficient and diet is not restricted. The positive relationship between circulating 3-hydroxybutyrate and NEFA observed here provides additional evidence toward fat-based ketogenesis (Fig. 4c–g). Although the ketogenic state is generally associated with a fasting state, we believe that the high levels of ketone bodies found in the blood of breast-fed infants uniquely supplies substrate for the rapidly growing brain. It has been previously hypothesized that the fatty acid oxidative activity in the brain is low to prevent generation of oxidative stress^29^. While glucose transport into, and utilization in, the brain during the breast-fed period is lower than in the adult state^30^, the brain can take up and metabolize ketone bodies in proportion to blood levels^30–32^. As there is no significant intracellular pool, circulating 3-hydroxybutyrate (the primary ketone body) accurately reflects the levels of brain 3-hydroxybutyrate^30^. Ketone bodies not only provide an alternative source of fuel for the brain (other than glucose), they serve as building blocks for endogenous synthesis of cholesterol and long chain fatty acids that are important for the developing brain^30^. Utilization of 3-hydroxybutyrate for biosynthesis of amino acids by the developing brain has shown to be more effective than that of glucose in rats^30,33^. Furthermore, a signaling role for 3-hydroxybutyrate at the cellular level of the brain and neurons has been recognized^34,35^. Although the difference was not significant, EF infants exhibited a trend toward higher levels of 3-hydroxybutyrate compared to SF infants (SI Fig. 1, Fig. 3c).

### “Metabolic stress” from a higher degree of protein utilization

The protein requirements of infants decline relatively rapidly during the first few months of life. Indeed, protein concentration in human milk declines as lactation continues^36,37^. In contrast, infant formula is not adjusted to account for the time-dependent change in protein requirement and contains markedly higher total protein content than breast milk. The “Early Protein Hypothesis” proposed that higher protein and nutrient supplies in the postnatal period may enhance the secretion of insulin and insulin-like growth factor I (IGF-I)^38^ as well as higher concentrations of circulating amino acids and urea^7^. Breast-fed infants have been shown to have lower circulating levels of IGF-1, essential amino acids (especially BCAA), urea and C-peptide in comparison to formula-fed infants^8,39^. Reducing the protein levels in infant formula has led to a mixed result of IGF-I levels^8,39–41^, although the results regarding decreased circulating levels of BCAA^8,10^ and urea^8,41^ are more consistent.

**Figure 6:**
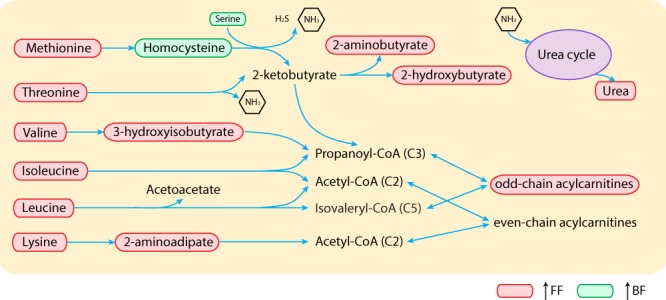
The amino acid catabolism pathway differs in breast-fed (BF) and formula-fed (FF) infants.

Following feeding, the postprandial peak of circulating amino acids is found mostly between 30–60 min, where circulating BCAAs, lysine, methionine, proline, threonine, and tyrosine exhibit the highest postprandial increase^9,42,43^. For both term and preterm BF infants, the clearance of diet-derived amino acids takes approximately 120 min^9,42,43^, whereas depending on the protein content and whey:casein ratio, elevated amino acids in the circulation of FF infants can still be detected at 120 min^9^, 180 min^42^, and 210 min post-meal^42^ in those consuming formula containing 15 g, 20 g or 30 g protein /L, respectively. Although the infant formulas used in the present study had a relatively low protein content (SF: 12.7 g protein /L, EF: 12 g protein /L), higher levels of circulating BCAAs, threonine, methionine, their degradation intermediates, and urea were still observed in FF compared with BF infants (Figs 1e, 3b and 6). We speculate that the elevated amino acid profile of FF infants at approximately 160–170 min post-meal (SI Table 4) is in part due to slow amino acid clearance. Serum urea, a marker in infants that does not fluctuate postprandially^9^, was higher in the FF infants when no or low amounts of complementary food were introduced (Figs 1e and 3b). This may be due to the elimination of excess nitrogen through insulin-mediated amino acid catabolism.

Catabolism of excess amino acids contributes to synthesis of propionyl-CoA (from methionine, threonine, valine and isoleucine), isovaleryl-CoA (from leucine), and acetyl-CoA (from glutamine, isoleucine, leucine and lysine) (Fig. 6). Since acetyl-CoA can also be synthesized from even-chain fatty acids, glucose, and acetoacetate, in concert with elevated amino acids, the increase in circulating acylcarnitine C3 observed here (Fig. 5b) potentially reflects promotion of fatty acid and triglyceride synthesis under higher insulin levels. Plasma odd-chain acylcarnitine species, including C3, C5, C5-OH and C5:1, have previously been found to be higher in 6-month-old infants consuming a high protein (20.5 g protein/L) compared with those consuming a lower protein (12.5 g protein/L) formula^10^. Here, the difference in acylcarnitine C3 between EF (12 g protein/L) and SF (12.7 g protein/L) infants was not significant (Fig. 5b), which is likely due to the small difference in protein content between the two formulas.

Tryptophan has long been considered the limiting amino acid in infant formula due to low bioavailability from cow’s milk protein^44^. If estimated from elemental formula, the tryptophan requirement would need to be nearly doubled to approximate the amount taken up from human milk^45^. This likely has to do with the differences in protein quality, digestibility, bioavailability and interaction with food matrices. Total milk tryptophan in human milk changes over lactation, ranging from approximately 200 to 400 mg/L^46^. In the present study, tryptophan concentrations were 250.8 and 228 mg/L for SF and EF, respectively, which is at the lower end of that in human milk. Nonetheless, a higher level of circulating tryptophan in FF compared to BF infants was observed only in GC-MS (Fig. 3b), but not in NMR data (Fig. 1e). This difference may be explained by the difference in sample preparation method, as protein precipitation using methanol (MS data) disrupts amino acids bound to serum proteins^47^.

### Complementary food impacts fat-based metabolism

In the present study, introduction of complementary food before 6 months was observed to impact metabolism. As complementary food was introduced, the BF- or FF- specific phenotype difference was not as distinguishable. This can be partly explained by a reduction of the total intake of breast milk or infant formula (high fat and low carbohydrate) and introduction of additional carbohydrate into the diet. Through the process of supplying more carbohydrate into the diet, infants progressively move toward utilization of carbohydrates as the main energy source, and eventually, the brain takes up and utilizes less ketone bodies, switching to using glucose as the predominant fuel^48,49^. Here, we observed a higher level of serum glucose in FF infants compared to BF infants at 12 months (Figs 1e and 2d). We speculate this difference may be due to a decreased ability to regulate glucose homeostasis; however, more studies are needed to confirm this observation. Furthermore, higher levels of BCAAs were observed in the SF infants when compared to EF and BF infants only if no or a low amount of complementary food was introduced before 6 months of age (Fig. 2a–c). This prolonged dietary impact was also observed by Karlsland Åkeson *et al*. who showed that by 12 months of age, infants fed a casein-predominant, high-protein formula (whey:casein, 18:82, 18 g protein/L) had higher circulating BCAAs, tyrosine, methionine and creatinine compared to exclusively or partially BF infants^11^.

### Signature of phospholipid supplementation in the serum metabolome and its implications for brain development

Depending on the stage of lactation, about 62–80% of phospholipids in human milk is comprised of phosphatidylethanolamine, phosphatidylcholine and sphingomyelin with primarily palmitic (16:0), stearic (C18:0), oleic (C18:1), and linoleic (C18:2) acids in their fatty acid tail^50^. Although phospholipids are present in infant formula, their concentrations vary amongst different formula manufacturers. While the proportion of sphingomyelin is higher than that of phosphatidylcholine in human milk^50^, in general, infant formulas contain a higher proportion of phosphatidylcholine than sphingomyelin due to the addition of soy lecithin as a stabilizer and emulsifier^50^.

Sphingomyelin is digested to ceramide by biliary secretion of alkaline sphingomyelinase^51^. Cholesterol esterase or bile-salt stimulated lipase (CEL/BSSL) can further hydrolyze ceramide to release fatty acids and sphingosine^52^. Phospholipase A2 (PLA2), secreted into the duodenum and expressed in the lungs and kidney, can hydrolyze the *sn*-2 position of phosphatidylcholine^53^. Furthermore, although dietary triglycerides can be partitioned into phospholipids after being digested and absorbed into enterocytes^54^, direct incorporation of dietary triglyceride fatty acids into the fatty acid tails of phospholipids has not been observed in infants^55^. Indeed, with no modification of triglyceride content in the two formulas in this study, we observed a higher level of LysoPC (14:0/0:0), (16:0/0:0), (17:0/0:0), (18:1/0:0), (18:2/0:0), and (18:3/0:0) in infants who consumed the phospholipid enriched EF compared to the SF group (Figs 3c and 5d,e). In particular, higher levels of circulating LysoPC (20:5) was observed in the BF infants, which is likely from phosphatidylinositol that contains a high level of eicosapentaenoic acid (EPA) in human milk^50^.

Serum levels of *myo*-inositol, choline, and metabolites from choline degradation (betaine and dimethylglycine) exhibit an increasing trend in infants receiving EF compared to infants consuming SF (SI Figs 1, 3c). Serum choline levels are higher in BF infants compared with FF infants^56^ and its concentration may be positively correlated with the choline content of breast milk^56^. Serum choline was also elevated in Peruvian infants supplemented with bovine MFGM fortified complementary food compared to control^57^. Moreover, dietary phospholipids supply a source of long-chain polyunsaturated fatty acids (LCPUFA). LCPUFA-containing lysophospholipids, choline, serine, and m*yo*-inositol in circulation, can be transported through the blood-brain barrier^58–61^ and serve as important substrates to support optimal phospholipid synthesis in the rapidly developing brain^58,62^. This may partly explain the difference in neurocognitive development that we previously reported^15^ (Table 1), and was reported by others in piglets^63^ and mice^64^, suggesting that dietary supplementation of phospholipid-enriched bovine MFGM may be a viable approach to promote brain development in formula-fed infants.

### Limitations

One technical limitation in the current study is the use of the non-targeted MS screening method, which identifies and quantifies metabolites without validation using stable isotope dilution. Furthermore, this study followed infants born between 2008 and 2011, and about 66% of those infants consumed >60 kcal (approximately 10% of daily energy requirement) from complementary feeding. The proportion of complementary feeding and type of food introduced may vary when comparing this population to a population from a different region.

## Conclusions

Before complementary food was introduced, different feeding modes led to a shift in metabolism based on the utilization of energy substrate (protein or fat). BF infants showed a higher rate of fatty acid oxidation and elevated medium- and long-chain acylcarnitines. FF infants had a lower efficiency of amino acid clearance and a higher rate of amino acid catabolism, odd-chain fatty acid synthesis through the C3-acylcarnitine precursor, and higher circulating urea. With increased complementary feeding, differences between BF and FF infants decreased, indicating a transition toward adaptation to a carbohydrate-dominant diet. In line with the previously reported cognitive improvement^15^, MFGM supplemented in infant formula has the potential to improve circulating lysophospholipids to a profile similar to that observed in BF infants. Although not significant through hypothesis testing, a trend toward lower circulating amino acids and higher choline, betaine and ketone bodies were observed in the EF infants, suggesting these infants are becoming more metabolically similar to BF infants. This work suggests that more research on MFGM supplementation in the field of pediatric and child endocrinology is warranted.

## Methods

### Characteristics of study population and data exclusion

This double-blinded, parallel randomized controlled trial took place at Umeå University Hospital, Umeå, Sweden after approval by the Regional Ethical Review Board in Umeå (Dnr 07–083 M) and obtaining both oral and written informed consent from the parents/caregivers before inclusion. The study was registered at clinicaltrials.gov (NCT00624689), and was conducted in accordance with the Declaration of Helsinki and Good Clinical Practice guidelines.

In total, 240 infants (BF, n = 80; SF, n = 80; EF, n = 80) and their parents were recruited in the original randomized controlled trial. A detailed description of sample size estimation, procedure for randomization and blinding, and exclusions is provided elsewhere^15^. Inclusion criteria included infants less than 2 months of age with a gestational age at birth of 37–42 weeks, birth weight of 2.5–4.5 kg, absence of chronic illness and either exclusively breast-fed or formula-fed prior to enrollment. Formula-fed infants were stratified for sex and randomly assigned to consume either EF or SF using a computerized randomization tool in blocks of 8. Parents and staff were blinded until all interventions were completed. Rates of dropout and noncompliance are reported elsewhere^15^.

#### The longitudinal subset (assessment at 2, 4, 6 and 12 months of age)

A subset of 30 infants (15 males and 15 females) from each dietary group were randomly selected for this subset in which the serum metabolome was assessed using NMR. Out of the 90 infants selected, samples from several infants were excluded from analysis: n = 2 infants from the formula-fed group who stopped consuming study formula before the end of study, n = 1 infant who had no food dietary record, n = 3 breast-fed infants who were heavily mixed-fed, n = 4 infants from the formula-fed group who consumed non-study formula, and n = 3 infants who had a record of antibiotics use. Numbers of samples from each intervention and time point are summarized in SI Tables 2 and 3.

#### The cross-sectional subset (assessment at 6 months of age)

Plasma samples from 212 infants at 6 months were subjected to GC- and LC- MS untargeted metabolomics analysis. For the samples assessed using GC-MS: 21 samples at the end of the batch were destroyed due to a crash of the instrument and 1 sample failed the QC. For the remaining 190 samples, samples from several infants were excluded from analysis: n = 4 infants from the formula-fed group who consumed non-study formula and either stopped or had no record of study formula consumption, n = 8 breast-fed infants who were mixed-fed, n = 1 infant who only fasted for 20 min, n = 4 infants who had a record of antibiotics use, and n = 17 infants who had no food dietary record for a total of 156 samples. For samples assessed using LC-MS: 1 sample failed QC. For the remaining 211 samples, samples excluded from analysis were from: n = 5 infants from the formula-fed group who consumed non-study formula and either stopped or had no record of study formula consumption, n = 8 breast-fed infants who were mixed-fed, n = 1 infants only fasted for 20 min, n = 8 infants who had a record of antibiotics use, and n = 17 infants who had no food dietary record for a total of 172 samples. Numbers of samples from each intervention used for analysis are summarized in SI Table 3.

### Study formula

Infants in the SF group consumed BabySemp1 (Semper AB, Sundbyberg, Sweden). Infants in the EF group consumed a formula modified from BabySemp1 with the addition of bovine MFGM-enriched whey protein concentrate (Lacprodan® MFGM-10; Arla Foods Ingredients, Viby J, Denmark). Both study formulas (powder, ready to feed) were provided to the parents with preparation instructions.

Lacprodan® MFGM-10 is manufactured by obtaining the retentate from microfiltration (0.1–0.2 µ) of whey during the yellow cheese production process^65^. MFGM-10 contains bovine MFGM fragments and is composed of 72 ± 2% protein, max. 2% lactose, 1.5% sialic acid, 18 ± 2% lipids including 6 ± 1% phospholipids and 0.6% cholesterol. The highest phospholipids in MFGM-10 is sphingomyelin (1.8%), followed by phosphatidylcholine (1.7%) and phosphatidylethanolamine (1.5%). Lacprodan MFGM-10 was incorporated to account for 4% (wt:wt) of the total protein in the EF.

The macronutrient composition of the EF and SF has been described previously^15^ and also available in SI Table 5. The majority of micronutrients remained the same between the SF and the EF with a few exceptions: concentration of folate (62 and 55 µg/L), vitamin B12 (0.56 and 0. 59 mg/L) and vitamin B6 (2.2 and 2.7 µg/L) were slightly different between EF and SF.

### Estimation of nutrient intake

Dietary intake was recorded by the parents through a 3-day food dietary record every month during 3 consecutive days at 2, 3, 4, 5 and 6 months of age. The intake of infant formula and complementary food was recorded by volume or weight by the parents using personal household measures and kitchen utensils. The nutrient intake of each complementary food was calculated according to the Swedish National Food Agency Database and food labels. Parents were encouraged to provide a small taste portion of complementary food between 4 and 6 months of age to complement breast milk and infant formula.

### Blood collection

Venous blood was collected in EDTA and SST tubes drawn at each visit, on average, 160–170 min, or at least 95 min after a meal. The fasting time for each group is summarized in SI Table 4. The exact ages for blood draw are available in SI Table 6. Plasma at baseline, 4, and 12 months were obtained by centrifugation at 1,300 × g for 10 mins and frozen. At 6 months, plasma and erythrocytes were separated by centrifugation at 2,000 × g for 10 min at 4 °C and plasma was frozen.

### Metabolomics analysis

Detailed methods for sample preparation, data acquisition and processing for both NMR-based and MS-based metabolomics analysis are available in the Supplementary methods.

### Statistical analysis

Statistical computing and graphical generation were performed using R programing environment. All plots were generated using *ggplot2*. Generalized log transformation (defined as *log(y* + *sqrt(y*^*2*^ + *lambda))*) was applied to all metabolomics data where *lambda* is 1. Principal component analysis was computed using *prcomp* function where each variable was centered by subtracting to the variable means (*center* = *True*), but not scaled by standard deviation (*scale*. = *False*).

The random forest model was built using the *randomForest* function from *randomForest* package where number of tree (*ntree*) was 1001, *mtry* was set as 8, which was determined as the square root of number of metabolites applied to the model. For each time point, a confusion matrix was generated corresponding to the OOB (out-of-bag) error that reflects the accuracy of the model (or number of cases in each category that were guessed correctly). The probability in the y-axis of Fig. 1c was calculated as the odds of predicting each intervention group correctly.

Significance between groups was evaluated using the Kruskal-Wallis test (*kruskal*.*test* function). For the pairwise comparison between BF, SF and EF groups, Kruskall-Wallis test followed by Dunn’s post hoc test (*posthoc*.*kruskal*.*dunn*.*test* function from *PMCMR* package) was applied where the multiple comparison was adjusted by Bonferroni correction (*p*.*adjust*.*method* = “*bonferroni”*). To counteract the problem of multiple comparison across all metabolites tested, the *p*-value from Kruskall-Wallis test or from Dunn’s post hoc test were further adjusted by False Discovery Rate (*p*.*adjust(*, *method* = ‘*fdr’)*). The overall level of significance was set at p < 0.05.

Effect size between BF and FF and between SF and EF was evaluated using Cliff’s delta (δ) statistic, a robust nonparametric alternative to Cohen’s d, using the *cliff*.*delta* function from *effsize* package. 95% confidence interval of each computed Cliff’s delta was estimated assuming a normal distribution (*use*.*normal* = *TRUE*). The magnitude was assessed using the thresholds suggested by Romano *et al*.^66^ where |δ| < 0.33 corresponds to small, |δ| < 0.474 corresponds to medium, otherwise large.

Linear regression analyses were performed to determine the association between each free fatty acid and 3-hydroxybutyrate. The square of Pearson’s correction coefficient (*r*^2^) was computed using *cor(method* = “*pearson”)* to evaluate the strength of correlation.

## Electronic supplementary material

Supplementary Material

## Data Availability

Data including raw metabolomics data (NMR and MS spectra) and relevant metadata are available from the corresponding author on request.
