# Publisher Correction: Metabolic phenotype of breast-fed infants, and infants fed standard formula or bovine MFGM supplemented formula: a randomized controlled trial

**DOI:** 10.1038/s41598-019-48858-y

**Published:** 2019-08-22

**Authors:** Xuan He, Mariana Parenti, Tove Grip, Magnus Domellöf, Bo Lönnerdal, Olle Hernell, Niklas Timby, Carolyn M. Slupsky

**Affiliations:** 1 Department of Nutrition, University of California Davis, One Shields Ave, Davis, CA 95616 USA; 2 Department of Food Science and Technology, University of California Davis, One Shields Ave, Davis, CA 95616 USA; 3 Department of Clinical Sciences, Pediatrics, SE 901 85 Umeå University, Umeå, Sweden

Correction to: *Scientific Reports* 10.1038/s41598-018-36292-5, published online 23 January 2019

In the original version of this Article, the author Carolyn M. Slupsky was incorrectly indexed. This error has now been corrected.

