# Supplementary material for: Metabolic phenotype of breast-fed infants, and infants fed standard formula or bovine MFGM supplemented formula: a randomized controlled trial
Source: Sci Rep. 2019 Jan 23;9:339. doi: 10.1038/s41598-018-36292-5 (PMC6344597; doi:10.1038/s41598-018-36292-5)

Supplementary information

Metabolic phenotype of breast-fed infants, and infants fed standard formula or bovine MFGM supplemented formula: a randomized controlled trial

Xuan He, Mariana Parenti, Tove Grip, Magnus Domellöf, Bo Lönnerdal, Olle Hernell, Niklas Timby, Carolyn M. Slupsky

Table of contents

- **Supplementary Methods**
  - Quantitative NMR-based metabolomics assessment
    - Sample preparation
    - NMR acquisition, data processing and quantification
  - Untargeted MS-based metabolomics assessment
    - Sample preparation
    - GC-MS analysis: data acquisition and processing
    - LC-MS analysis: data acquisition and processing
- **Supplementary Tables**
  - **SI Table 1.** Infant serum or plasma metabolites identified using NMR, LC-MS and GC-MS.
  - **SI Table 2.** Number of samples in each intervention group.
  - **SI Table 3.** Number of samples from breast-fed (BF) or formula-fed infants (Standard Formula, SF; Experimental Formula, EF) in a subset depending on the complementary food intake, with either > 60 kcal of daily energy or < 60 kcal of daily energy from complementary food.
  - **SI Table 4.** Fasting time (min) prior to blood drawing.
  - **SI Table 5.** Macronutrient contents of standard and experimental formula
  - **SI Table 6.** The exact ages (days) when blood was drawn.
- **Supplementary Figure**
  - **SI Figure 1**. NMR quantified serum metabolites significantly different between formula-fed infants who consumed standard formula (SF) (red) or experimental formula (EF) (orange). The 95% confidence interval was estimated using the normal distribution.

Supplementary Methods

Quantitative NMR-based metabolomics assessment

Sample preparation. Serum samples were filtered through a 3 kDa molecular weight ultracentrifugal filter (Amicon ultracentrifugal filter, Millipore, Billerica, MA) to remove insoluble lipid particles and proteins. The volume of each filtrate was carefully measured and the appropriate amount of Mili-Q water (Milipore, Billerica, MI) was added to each filtrate to ensure a final volume of 199 µL. 8 µL of potassium phosphate buffer (1M, pH 6.1) was added, and the sample pH was adjusted to 6.80 ± 0.08 (average ± standard deviation). 23 µL of an internal standard (5 mM DSS-d6) containing 0.2% NaN_3 in 99.8% D_2O was added to inhibit microbial growth and ensure NMR locking respectively. 180 µL aliquots were subsequently transferred to 3 mm Bruker NMR tubes (Bruker, Brillerica, MA) and stored at 4 °C until spectral acquisition.

NMR acquisition, data processing and quantification. ^{1}H NMR spectra were acquired at 298K using the NOESY ^{1}H presaturation experiment ('noesypr1d') on a Bruker Avance 600 MHz NMR spectrometer (Bruker BioSpin, Germany) equipped with a SampleJet autosampler (Bruker BioSpin, Germany). Data were acquired using 32 transients and 8 dummy scans over a spectral width of 12 ppm with a total acquisition time of 2.5 s. Water saturation was applied during relaxation delay (2.5 s) and mixing time (100 ms). The resulting spectra were Fourier transformed with zero filling to 128 k data points and the Free Induction Decays (FIDs) were transformed with an exponential apodization function corresponding to a line-broadening of 0.5 Hz. Spectra were manually phased and baseline corrected using Chenomx NMR Suite v8.3 (Chenomx Inc, Edmonton, Alberta, Canada). Quantification of each metabolite was assigned manually using Chenomx Profiler based on the established method of targeted profiling [1].

Untargeted MS-based metabolomics assessment

Plasma from infants at 6 months of age were analyzed for the presence and relative quantity of metabolites using GC-MS and LC-MS at the Swedish Metabolomics Centre, Umeå, Sweden.

Sample preparation. Sample preparation was performed according to A et al.[2]. In detail, 900 µL of extraction buffer (90/10 v/v methanol:water) that included a mixture of internal standards for both GC-MS and LC-MS was added to 100 µL of plasma. Internal standards for GC-MS analysis included [$^{13}C_5$]-proline, [D_4]-succinic acid, [D_4]-salicylic acid, [$^{13}C_4$]-α-ketoglutarate (methoxymated derivatives), [$^{13}C_5$,^{15}N]-glutamic acid, [D_4]-putrescine, [1,2,3-$^{13}C_3$]-myristic acid, [$^{13}C_6$]-Glucose, [$^{13}C_4$]-hexadecanoic acid, [$^{13}C_{12}$]-sucrose and [D_7]-cholesterol. Internal standards for LC-MS analysis included [$^{13}C_9$]-phenylalanine, [$^{13}C_3$]-caffeine, [D_4]-cholic acid, [D_8]-arachidonic Acid, and [$^{13}C_9$]-caffeic Acid. Samples were shaken at 30 Hz for 3 min in a mixer mill then left on ice to allow proteins to precipitate. The samples were subsequently centrifuged for 10 min (4 °C, 14k rpm). For each sample, two 200 µL aliquots of the supernatant were transferred to microvials and each was dried using a speed-vac concentrator to generate the samples for GC-MS and LC-MS. A QC (Quality Control) pool was made by combining the left over extract from 41 randomly selected samples. 200 µL aliquots were prepared from the QC pool and run as quality control throughout the GC-MS and LC-MS analyses.

For GC-MS samples, 30 µL of methoxyamine (15 µmol/L) in pyridine was added to each GC vial followed by vigorously vortexing for 10 min. Methoxymation was carried out at room temperature for 16 h. 30 µL of MSTFA with 1% TMCS as a catalyst was added followed by 10 min vortex mixing. After silylation for 1 h, 40 µL of heptane was added to each GC vial.

For LC-MS samples: samples was resuspended in 20 µL (1:1 methanol:water).

GC-MS analysis: data acquisition and processing. GC-MS analysis was performed on an Agilent 6890 gas chromatograph equipped with a CTC Combi Pal autosampler (CTC Analytics AG, Switzerland) and coupled to Pegasus III TOFMS (Leco Corp., St Joseph, MI) as previously described[2]. The GC was operated in splitless mode with 1 µl of derivatized sample injected. The separation of compounds was achieved by utilizing a 10 m x 0.18 mm fused silica capillary column with a chemically bonded 0.18 µm DB 5-MS UI stationary phase (J&W Scientific) at 270 °C injector temperature. The purge time was 60 sec at a purge flow rate of 20 mL/min. The column temperature was initially kept at 70 °C for 2 min and then increased from 70 to 320 °C at 30 °C per min, where it was held for 2 min. Helium was used as carrier gas at a constant flow rate of 1 mL/min through the column. The transfer line temperature and ion source temperature were set at 250 °C and 200 °C, respectively. The mass spectra were recorded between 50 to 800 m/z at a rate of 30 spectra/sec throughout all samples at 70 eV electron energy with an ionization current of 2.0 mA and 1500-2000 V detector voltage. The acceleration voltage was applied after a solvent delay of 150 seconds.

After data acquisition, all non-processed MS-files were exported from ChromaTOF software (LECO) into MATLAB R2016a (Mathworks, Natick, MA, US). Baseline correction, chromatogram alignment, data compression and multivariate curve resolution were performed using custom scripts developed at the Swedish Metabolomics Centre and run on Matlab. Peak detection and compound identification was performed by comparing retention index and mass spectra with reference spectra[3] using the NIST spectral search program 2.0 (NIST/EPA/NIH Mass Spectral Library). Annotation of mass spectra was based on reverse and forward searches in the library. Masses and ratio between masses indicative of a derivatized metabolite were noted, and if the mass spectrum indicated a high probability for a particular metabolite and the retention index between the sample and the library for the suggested metabolite was ± 5, the deconvoluted peak was identified.

LC-MS analysis: data acquisition and processing. LC-MS analysis was performed on an Agilent 1290 Infinity UHPLC-system (Agilent Technologies, Waldbronn, Germany) interfaced with an Agilent 6540 Q-TOF mass spectrometer equipped with a jet stream electrospray ion source, operating in positive or negative ion mode.

2 µL of sample was applied onto an Acquity UPLC HSS T3 column (2.1 x 50 mm, 1.8 µm particle) in combination with a Van Guard precolumn (2.1 mm x 5mm, 1.8 µm particle, Waters Corporation, Milford, MA, USA) at 40 °C. The chromatographic separation was performed using 0.1% formic acid in water (mobile phase A) and 0.1 % formic acid in acetonitrile-isopropanol (75:25, v/v) (mobile phase B) under linear gradient condition. Under a flow rate of 500 µL/min, the compounds were eluted with a linear gradient consisting of 0.1-10% B over 2 min. B was then increased to 99% over 5 min and held at 99% for 2 min. The column was then returned to the initial conditions (0.1% B) for 0.3 min. To reduce re-equilibration time, the flow-rate was increased to 800 µL/min for 0.5 min, and held at this rate for 0.9 min, after which the flow-rate was reduced to 500 µL/min for 0.1 min before the next injection.

Analysis was performed in both polarities, positive and negative modes, in order to determine as many compounds as possible that have either basic or acidic characteristics. The settings of both modes were kept identical, with the exception of the capillary voltage. The instrument was operated at +4000 V capillary voltage in positive ion mode and -4000 V in negative ion mode, 40 psig nebulizer pressure, 300 °C gas temperature, 8 L/min drying gas flow, 0 V collision energy, 350°C sheath gas temperature and 11 L/min sheath gas flow. The nozzle, fragmentor, skimmer and octopole voltages were 0 V, 100 V, 45 V and 750 V respectively. Mass spectra were recorded across the measured range from 70 to 1700 m/z in centroid mode at 4 scans/sec. Blanks, diluted samples, and quality control samples were analyzed at the middle and the end of the sequence to control system stability.

Drifts in mass accuracy of the TOF were corrected by continuous infusion of two internal mass calibrants at a flow rate of 50 µL/min: purine at m/z 121.05 and m/z 119.03632 and HP-0921 [Hexakis(1*H*, 1*H*, 3*H*-tetrafluoro-pentoxy)phosphazene] at m/z 922.0098 and m/z 966.000725 for positive and negative mode, respectively.

Mass spectrum deconvolution was performed using Agilent Masshunter Profinder version B.08.00 (Agilent Technologies Inc. Santa Clara, CA, USA). Processing was performed in both targeted and untargeted fashion. For targeted profiling, a pre-defined list of metabolites commonly found in plasma and serum was searched using the Batch Targeted feature extraction in Masshunter Profinder. An in-house LC-MS library built by authentic standards run on the same system with the same chromatographic and mass spectrometer settings were used for targeted processing. Identification of metabolites was based on MS, MSMS, and retention time information. For untargeted data, the pooled QC-samples were processed using the Batch Recursive Feature Extraction algorithm in Masshunter Profinder. After exporting cef-files of all processed QC-samples, the extracted features were matched using Mass Profiler Professional 13.0™ (Agilent Technologies Inc. Santa Clara, CA, USA) resulting in a combined recursion file. This file was imported into Masshunter Profinder and used for Batch Targeted Feature Extraction on all samples.

References:

1 Weljie, A. M., Newton, J., Mercier, P., Carlson, E. & Slupsky, C. M. Targeted profiling: quantitative analysis of 1H NMR metabolomics data. *Anal Chem* **78**, 4430-4442 (2006).

2 A, J. *et al.* Extraction and GC/MS analysis of the human blood plasma metabolome. *Anal Chem* **77**, 8086-8094 (2005).

3 Schauer, N. *et al.* GC-MS libraries for the rapid identification of metabolites in complex biological samples. *FEBS Lett* **579**, 1332-1337 (2005).

Supplementary Tables

SI Table 1. Infant serum or plasma metabolites identified using NMR, LC-MS and GC-MS.

Category	Compound	NMR	LC-MS		GC-MS
			Positive ion mode	Negative ion mode	
Sugars	Fructose	√			√
	Glucose	√			√
	Mannose	√			
	Galactose	√			
	Xylose	√			√
	Maltose				√
Sugar alcohols	Arabitol				√
	myo-Inositol	√			√
Sugar acids	Galacturonate				√
Amino acids	2-aminobutyrate	√			√
	Alanine	√			√
	Arginine	√			
	Asparagine	√			√
	Asparate	√			
	β-Alanine	√			
	Citrulline				√
	Cysteine				√
	Glutamate	√			
	Glutamine	√			√
	Glycine	√			√
	Histidine	√			√
	Lysine	√			√
	Methionine	√	√		
	Ornithine	√			
	Phenylalanine	√	√	√	√
	Proline	√			√
	Taurine	√			√
	Threonine	√			√
	Tryptophan		√	√	
	Tyrosine	√	√	√	√
	Valine	√			√

Category	Compound	NMR	LC-MS		GC-MS
			Positive ion mode	Negative ion mode	
	Leucine	√	√ (cannot differentiate)	√ (cannot differentiate)	√
	Isoleucine	√			√
**Peptide**	γ-Glu-Leu		√		
**Amino acid derivatives**	2-amino-adipic acid				√
	2-oxoglutarate	√			√
	2-Oxoisocaproate	√		√	√
	3-hydroxyisobutyrate	√			
	3-hydroxyisovalerate	√			
	3-methylhistidine	√			
	Asymetric dimethylarginine (ADMA)				√
	Betaine	√			
	Creatine	√			
	Creatinine	√			√
	Dimethylglycine	√			
	Ethanolamine	√			
	Hydroxyproline	√			
	Kynurenine		√		
	methylguanidine	√			
	Pyroglutamate	√	√	√	√
	Serine	√			√
	Urea	√			√
	Urocanoate	√			
**Ketones**	Acetone	√			
	Acetoacetate	√			
	3-hydroxybutyrate	√			√
**Vitamines/essential nutrients**	Alpha-tocopherol (Vitamine E)				√
	Ascorbate	√			
	Choline	√			

Category	Compound	NMR	LC-MS		GC-MS
			Positive ion mode	Negative ion mode	
	Pantothenate		√	√	
TCA cycle intermediates and energy metabolism	Citrate	√		√	√
	Fumarate	√			√
	Lactate	√		√	√
	Malate			√	√
	Pyruvate	√			
	Succinate	√			
Phosphatidylcholine and lysophosphatidylcholines (LysoPC)	LysoPC(14:0/0:0)		√	√	
	LysoPC(16:1/0:0)		√	√	
	LysoPC(17:0/0:0)		√	√	
	LysoPC(18:0/0:0)		√	√	
	LysoPC(18:1/0:0)		√	√	
	LysoPC(18:2/0:0)		√	√	
	LysoPC(18:3/0:0)			√	
	LysoPC(20:4/0:0)		√	√	
	LysoPC(20:5/0:0)		√	√	
	LysoPC(22:6/0:0)		√	√	
	1-palmitoyl-sn-glycero-3-phosphocholine				√
Hormone	Cortisol		√		
	Thyroxine		√		
Acylcarnitines	Acetylcarnitine (C2)	√	√		
	Propionylcarnitine (C3)		√		
	Butyrylcarnitine (C4)		√		
	Hexanoylcarnitine (C6)		√		
	Octanoylcarnitine (C8)		√		
	Decanoylcarnitine (C10)		√		
	Palmitoylcarnitine (C16)		√		
	Oleoylcarnitine (C18:1)		√		
Short-chain fatty acids	2-hydroxyisovalerate	√			
	Acetate	√			
	Butyrate	√			
	Formate	√			

Category	Compound	NMR	LC-MS		GC-MS
			Positive ion mode	Negative ion mode	
	Isobutyrate	√			
	Propionate	√			
Free fatty acids	Lauric acid (12:0)				√
	Palmitic acid (16:0)				√
	Stearic acid (18:0)		√		√
	Oleic acid (18:1)		√		√
	Linoleic acid (18:2)		√		√
	Arachidonic acid (12:4)		√		
	Docosahexaenoic acid (22:6)		√		
Host-microbes co-metabolites	1,2-propanediol (propylene glycol)	√			
	Dimethyl sulfone	√			
	Dimethylamine	√			
	Hippurate		√	√	√
	Trimethylamine N-oxide (TMAO)	√			
Metabolites of plant origin	4-hydroxyphenylacetic acid				√
	Caffeine		√		
	Gluconic acid				√
	Indole-3-acetate				√
	Proline betaine	√			
	Theobromine		√		
Nucleotides/nuclei acid metabolism	Hypoxanthine	√	√	√	
	Uric acid		√	√	√
	Uridine	√			
	Xanthine		√		
Cholesterol/bile acids	Cholesterol				√
	Glycocholic acid		√	√	
	Glycodeoxycholate/chenodeoxycholic acid glycine conjugate			√	
	Taurochenodeoxycholic acid			√	
Metabolites of drug/industry origin	1,5-anhydro-D-glucitol				√
	Acetaminophen (tylenol)	√	√		√
	Adipic acid				√
	Lidocaine				√
	Salicylic acid			√	
	Theophylline		√		
Alcohols	Ethanol	√			

Category	Compound	NMR	LC-MS		GC-MS
			Positive ion mode	Negative ion mode	
	Isopropanol	√			
	Methanol	√			
**Other organic compounds**	2-hydroxybutyrate	√			
	Adenosine-5-monophosphate				√
	cis-aconitate				√
	Glycerate				√
	Glycerol	√ (maybe contamination)			

SI Table 2. Numbers of samples in each group.

		Breastfed				Standard formula-fed				Experimental formula-fed			
Age (months)		**2**	**4**	**6**	**12**	**2**	**4**	**6**	**12**	**2**	**4**	**6**	**12**
The longitudinal subset	NMR data	25	18	24	26	23	20	22	24	24	21	27	27
The cross-sectional subset	GC-MS data			58				45				53	
	LC-MS data			58				54				60	

SI Table 3. Numbers of samples from breast-fed (BF) or formula-fed infants (Standard Formula, SF; Experimental Formula, EF) in a subset depending on the complementary food intake, with > 60 kcal of daily energy (with) or < 60 kcal of daily energy (without) from complementary food.

	Age (months)	Without complementary food subset			With complementary food subset		
		**BF**	**SF**	**EF**	**BF**	**SF**	**EF**
**NMR data**	**4**	17	18	20	11	9	8
	**6**	1	2	1	13	13	19
**GC-MS data**	**6**	21	13	16	37	32	37
**LC-MS data**	**6**	21	16	21	37	38	39

SI Table 4. Fasting time (min) prior to blood drawing. Values are expressed as mean ± standard deviation [minimum].

	Breastfed	Standard formula-fed	Experimental formula-fed	
**The longitudinal subset**				
Baseline	164.0 ± 33.9 [130]	189.3 ± 54.4 [120]	179.2 ± 34.1 [120]	NMR data
4 & 6 months of age (without complementary food)	158.2 ± 20.7 [125]	168.7 ± 29.7 [120]	168.6 ± 35.6 [125]	
4 & 6 months of age (with complementary food	170.7 ± 29.0 [120]	178.3 ± 46.1 [135]	166.0 ± 34.2 [120]	
12 months of age	171.0 ± 42.2 [95]	170.2 ± 31.5 [110]	188.9 ± 48.2 [135]	
**The cross-sectional subset**				
6 months of age (without complementary food)	152.1 ± 21.6 [105]	173.5 ± 43.9 [120]	154.4 ± 31.3 [95]	GC-MS data
6 months of age (with complementary food)	163.7 ± 29.6 [120]	174.7 ± 37.0 [120]	167.4 ± 39.1 [120]	
6 months of age (without complementary food)	152.1 ± 21.6 [105]	171.0 ± 40.5 [120]	161.4 ± 37.4 [95]	LC-MS data
6 months of age (with complementary food)	163.7 ± 29.6 [120]	174.1 ± 36.2 [120]	170.4 ± 41.9 [120]	

SI Table 5. Macronutrient contents of standard and experimental formula after preparation.

	**Standard formula**	**Experimental formula**
Energy (kcal/L)	660	600
Protein (g/L)	12.7	12
Casein (g/L)	5	3.5
Whey (g/L)	8	8.5
Carbohydrates/lactose (g/L)	74	60
Lipids (g/L)	35	35
SFAs (g/L)	13	13.5
MUFAs (g/L)	14	13.5
PUFAs (g/L)	6	6
Linoleic acids (g/L)	4.6	4.6
α-Linolenic acid (g/L)	0.7	0.7
Arachidonic acid (g/L)	0.15	0.15
DHA (mg/L)	90	90
Cholesterol (mg/L)	40	80
Phospholipids (mg/L)	300	700

SI Table 6. The exact ages (days) when blood was drawn. Values were expressed as mean ± standard deviation. BF: breastfed; SF: Standard formula-fed; EF: Experimental formula-fed.

	Breastfed	Standard formula-fed	Experimental formula-fed	
**The longitudinal subset**				
Baseline	48.9 ± 4.6 days	48.2 ± 8.0 days	44.1 ± 8.5 days	NMR data
4 months of age	121.4 ± 2.9 days	121.3 ± 2.4 days	121.2 ± 3.3 days	
6 months of age	182.9 ± 8.0 days	182.4 ± 6.3 days	181.7 ± 6.0 days	
12 months of age	359.0 ± 7.3 days	364.3 ± 9.0 days	364.2 ± 8.3 days	
**The cross-sectional subset**				
6 months of age	182.8 ± 7.3 days	184.9 ± 6.8 days	181.6 ± 6.1 days	GC-MS data
6 months of age	182.8 ± 7.3 days	183.7 ± 6.8 days	181.4 ± 6.0 days	LC-MS data

Supplementary Figure

<u>SI Figure 1</u>. NMR quantified serum metabolites significantly different between formula-fed infants who consumed standard formula (SF, red) or experimental formula (EF, orange). The 95% confidence interval was estimated using a normal distribution.

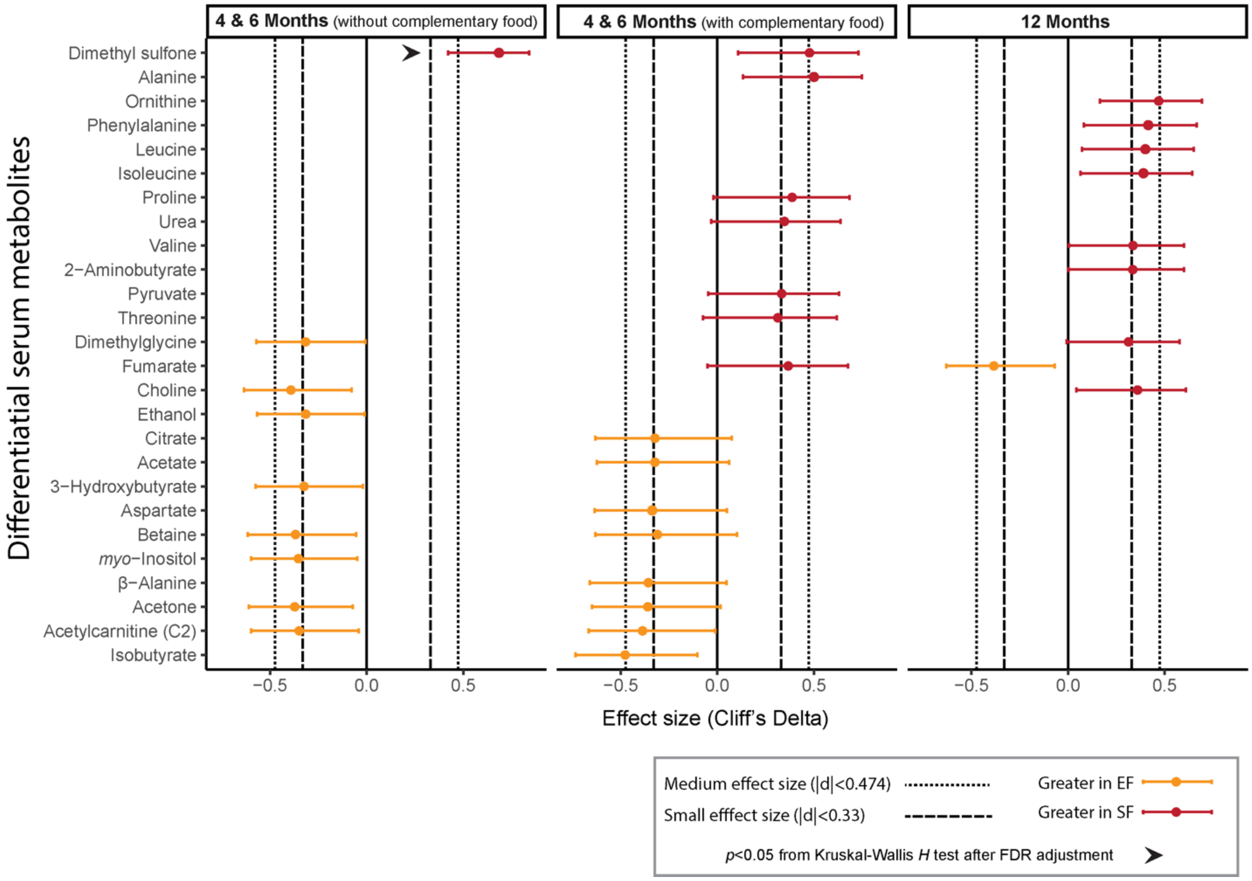

Supplement: Supplementary file 1 — Supplementary Material [file 41598_2018_36292_MOESM1_ESM.pdf]
